# British Medicinal Plants

**Published:** 1891-06

**Authors:** Alfred Heath

**Affiliations:** London, England


### BRITISH MEDICINAL PLANTS.

ALFRED HEATH, M. D., F. L. S., LONDON, ENGLAND.

### ORDER 25.—LEGUMINOSÆ. (CONTINUED.)

*Trifolium pratense* (Purple Clover).

Trifolium repens (White Clover or Dutch Clover).

Trifolium arvense (Haresfoot Trefoil).

The trifoliums have not been much used in medicine, and I only give the names of the three kinds that have been mentioned in homœopathic works. The white clover was at one time esteemed as a remedy in gout, inflammation, etc. The juice dropped into the eye was said to remove films, heat, and inflammation. It was also applied to the bites of adders and other venomous creatures. Dr. Allen's *Materia Medica* contains some short provings of the two first named.

### ORDER 26.—ROSACEÆ.

*Prunus spinosa* (Sloe, Blackthorn).—A form of *Prunus communis.* Common in our hedges, flowering before the leaves appear, in the early spring. The wood of the blackthorn is very hard, and elegant walking sticks are made from it, also that very handy instrument of torture our Irish brothers are so fond of using on each other, or any other, the shillalah, also called "illigant." The young leaves have been used to adulterate tea. The deep-red juice of the sloe is used to adulterate wine. So, possibly, the sloe may be in at the beginning of a quarrel as well as at the end of one. The juice of the berry is said to make famous marking ink, and it is said to be so indelible that no acid will take it out. It is also very purgative, so also are the flowers. Preparations made from the sloe have been used as gargles in enlargement of the tonsils and uvula, sore mouth and gums, loose teeth, etc. It has also been used as an astringent in hemorrhages.

There is a proving of this drug in Hering's *Guiding Symptoms.* It produces both diarrhœa and constipation, pains in the teeth as if they would be torn out, or, as if the teeth would be raised from the gums, and many other symptoms.

*Prunus Pádus* (Bird Cherry).—The kernel within the stone was the part used, and it was given as a remedy against *apoplexies, palsies,* and many other *nervous diseases.* The water distilled from it was in constant use as a remedy for children's diseases, but it fell into disuse, as, if not given carefully, or made too strong, it was found to *aggravate or occasion the disorder it was given to cure.* This is easily accounted for when we remember that many, if not all of the sub-order Amygdaleæ, to which Pruni belong, contain *Hydrocyanic acid.* There is no proving of *Prunus Pádus,* but the analogy will be seen when I come to *P. Laurocerasus.* It has cured intermittents, and been found useful in syphilis.

*Prunus Avium* (Wild Cherry).—Probably similar in character to the preceding. There is no proving.

*Prunus Laurocerasus,* or Laro-cerasus (The common Laurel or Cherry Laurel).—This shrub is not indigenous, but it is a native of Asia Minor and Persia. It was introduced into Europe about the middle of the sixteenth century, since which time it has managed to live and thrive in all parts of this country. Having had between three and four hundred years' residence with us, entitles it to be considered naturalized. The *young* leaves and buds collected in May or June are the best; they yield 6.33 grains of oil in one thousand, whereas, in July, when they have attained their full size, the yield sinks to 3.1 grains, and this goes on lessening until, at twelve months old, they only contain 0.6.

Linnæus informs us that in Switzerland this drug is commonly and successfully used in *pulmonary* complaints. Langrish mentions its efficacy in *agues.* Baylies found it particularly efficacious in *rheumatism, asthma,* and scirrhous affections. Hering's *Guiding Symptoms* tells us that this drug *produces* and has also cured loss of consciousness, vomiting, eyes turned up and fixed, pupils dilated, vanishing of sight, roaring and singing in the ears, trembling and twitching of the muscles of the face, distortion of the face, rush of blood to the head, moaning and groaning, dry almost constant cough, cough with evening aggravation and rapid sinking of vital forces, continuous night cough on lying down, threatening paralysis of the lungs, pains in the pleura, cough, with great amount of expectoration, mingled with clots of blood; great dyspnœa and sensation as if the lungs would not sufficiently expand, gasping, suffocating spells. It also produces chills and external coldness, coldness and shivering in the afternoon and evening, not relieved by external warmth, chill alternating with heat, sweat after eating, during and after heat, till toward morning. Also laming pains in the right shoulder-joint, pain as if sprained in the wrist-joint, painful stiffness of left side of neck, pains as if sprained in hip-joint, and a great many other symptoms affecting every part of the body.

*Spirœa Ulmária* (Meadow-sweet).—The root of this plant is said to be singularly effective in fevers; also recommended in disorders of the skin, scrofula, etc. The flowers are said to be alexipharmic and sudorific and anti-spasmodic, and good in malignant distempers, in fluxes of all kinds; it promotes sweating. It is also a good wound herb, and has been found good in inflammation of the eyes. There is a short proving of this drug in Dr. Allen's *Materia Medica.*

*Spirœa Filipendula* (Dropwort).—A very elegant spiræa found on our chalk hills. This plant was at one time officinal. It possesses astringent properties, and is also said to be lithontriptic, but is seldom used in practice now.

*Agrimonia Eupatoria* (Common Agrimony).—This plant has been recommended in jaundice, and has been found good in diabetes and incontinence of urine, bloody urine, spitting of blood, cleansing the skin. It has the reputation of healing all inward or outward wounds, bruises, gun-shot wounds, etc., bites of serpents, coughs, agues, etc. It is said to cure bloody flux. Made into an ointment it is good for old sores, cancers, and inveterate ulcers; it is said to draw forth thorns, splinters, nails, and other such things that have got into the flesh. It is useful in sprains and dislocations. There is no proving.

*Potentilla Anserina* (Silverweed).—The leaves of this plant are mildly astringent and possess corroborant qualities, but are mostly used by the country people. No proving.

*Potentilla Tormentilla* (Tormentil).—This plant is said to be useful in diarrhœas and dysenteries, especially when attended by fever, it is accounted alexipharmic. It is useful in hemorrhages from the nose, mouth, or womb, for looseness of the teeth and relaxation of the uvula. It is said to be a good medicine in small-pox, and if purging comes on in that disorder nothing excels it. It is good against spitting of blood, bleeding piles, bloody stools, or immoderate menses.

